# Chlorhexidine Gluconate in Pediatric Endodontic Treatment: A Scoping Review

**DOI:** 10.7759/cureus.80445

**Published:** 2025-03-12

**Authors:** Tran Thi My Hanh, Nguyen Duy Hieu, Vo Truong Nhu Ngoc, Le Kha Anh, Tran Phuong Thao, Dinh Nguyen My Hau, Dao Thi Hang Nga

**Affiliations:** 1 School of Dentistry, Hanoi Medical University, Hanoi, VNM; 2 Division of Research and Treatment for Oral and Maxillofacial Congenital Anomalies, School of Dentistry, Aichi Gakuin University, Nagoya, JPN

**Keywords:** chlorhexidine digluconate, endodontic treatment, "pediatric dentistry"[mesh]), primary teeth root canal, scoping review

## Abstract

Chlorhexidine digluconate (CHX) is widely recognized for its antimicrobial properties and potential use as an irrigant in endodontic treatment. However, its specific role in the treatment of primary teeth remains uncertain. This review aimed to evaluate the effectiveness of CHX in cleaning the root canal system in pediatric endodontic treatment. A total of 46 studies were initially identified from three databases: PubMed (5), ScienceDirect (2), and Cochrane (0). Following selection based on PRISMA-ScR 2018 guidelines, seven studies met all inclusion criteria and were included in the qualitative analysis. The findings indicate that CHX 2% demonstrates good antibacterial efficacy but lacks the ability to dissolve inorganic tissue. Additionally, its safety and effectiveness in dissolving organic tissue in primary teeth have yet to be thoroughly investigated. The minimum irrigation volume when using CHX is 2 ml per root canal, which ensures optimal effectiveness in bacterial elimination and improves treatment outcomes. While CHX shows promise as an endodontic irrigation solution for primary teeth, further research is necessary to fully understand its potential benefits and limitations.

## Introduction and background

According to the Global Disease Report of the World Health Organization 2016, oral diseases affect about 3.5 billion people, of which 486 million children suffer from tooth decay, which, if not treated thoroughly, will lead to pulp disease [1]. The root canal system of deciduous teeth is highly complex, characterized by features such as accessory canals, enlarged apical foramina, and the resorption of roots [2]. These complexities highlight the critical role of irrigation in effectively cleaning the root canal system of primary teeth. Given the unique anatomical and structural properties of the root canals in deciduous teeth, the choice of irrigation solution must achieve a delicate balance: it must effectively cleanse the canal system while ensuring it does not harm the surrounding tissues or the developing permanent tooth germ located beneath. Commonly, irrigants, such as saline and sodium hypochlorite, fall short of fulfilling the criteria for an ideal irrigant [3]. Saline is ineffective in dissolving tissue and lacks antibacterial properties, making it insufficient for thorough cleaning. On the other hand, sodium hypochlorite can dissolve organic tissue and possess antibacterial qualities essential for infection control [3]. However, its use comes with significant drawbacks; it is toxic to the surrounding tissues and can harm the unique root canal morphology of primary teeth. In summary, the irrigation process in treating deciduous teeth requires careful consideration of the chosen irrigant. It must ensure the effective removal of debris and bacteria and safeguard the health of surrounding tissues and the integrity of the primary tooth’s structure.

Chlorhexidine gluconate (CHX) is suggested as an effective irrigation solution for deciduous teeth [4]. CHX is a synthetic lipophilic cationic bi-guanide that interacts with phospholipids and lipopolysaccharides on the bacterial cell membrane and then penetrates the cell through a transport mechanism, changing cell osmosis, thereby having a bacteriostatic or bactericidal effect depending on the concentration. In addition, CHX also inhibits biofilm formation, is antifungal, and has low toxicity [5]. To provide a comprehensive update, this scoping review aimed to determine the cleaning efficacy of the root canal system in pediatric endodontics with chlorhexidine gluconate.

## Review

Methods

The study was written according to Preferred Reporting Items for Scoping Reviews [6].

Search Strategies

Two independent reviewers conducted this literature search on studies published through July 2024. Three databases were screened: PubMed (Medline), ScienceDirect, and the Cochrane Library. A search strategy was developed through search terms. The references cited in the included articles were also checked to identify other potentially relevant articles. Once articles from the databases were identified, they were imported into the reference manager website Covidence to remove duplicates.

The criteria for selecting articles for inclusion were predefined following the PCC format (Population/Patient: Endodontic treatment of primary teeth, Concept: cleaning root canal with chlorhexidine gluconate results, Context: World). We placed no restrictions on the study design, year of publication, or study location. We limited studies to those in the English language.

The exclusion criteria were any review studies. Any studies without English full-text were excluded.

Study Selection

Two authors independently assessed all document titles and abstracts to ensure a thorough initial review. Following this initial screening, complete copies of all potentially relevant studies were selected for comprehensive evaluation. This selection included studies that appeared to meet the established inclusion criteria and those that lacked sufficient data in their titles and abstracts to make a definitive judgment.

Subsequently, two reviewers independently and duplicatively assessed the full-text papers to determine their eligibility. In cases where disagreements arose regarding the eligibility of the included studies, these were resolved through discussion and consensus between the reviewers. A third reviewer was brought in to provide an objective evaluation if consensus could not be reached. Ultimately, only articles that met all of the specified eligibility criteria were included in the final analysis. This rigorous process ensured that the selected studies were both relevant and of high quality, contributing to the integrity of the overall research findings.

Data Extraction and Analysis

The data were evaluated based on tissue solubility and antibacterial properties, extracted using a standardized form. For antibacterial studies, data were gathered on the author, comparison group, sample size, test type, and main findings. Also, the following data from the smear layer removal study were retrieved: comparison group, sample size, Rome scale, and main findings. If some information was missing, the authors were contacted via e-mail to retrieve it.

Results

Study Selection

**Figure 1:**
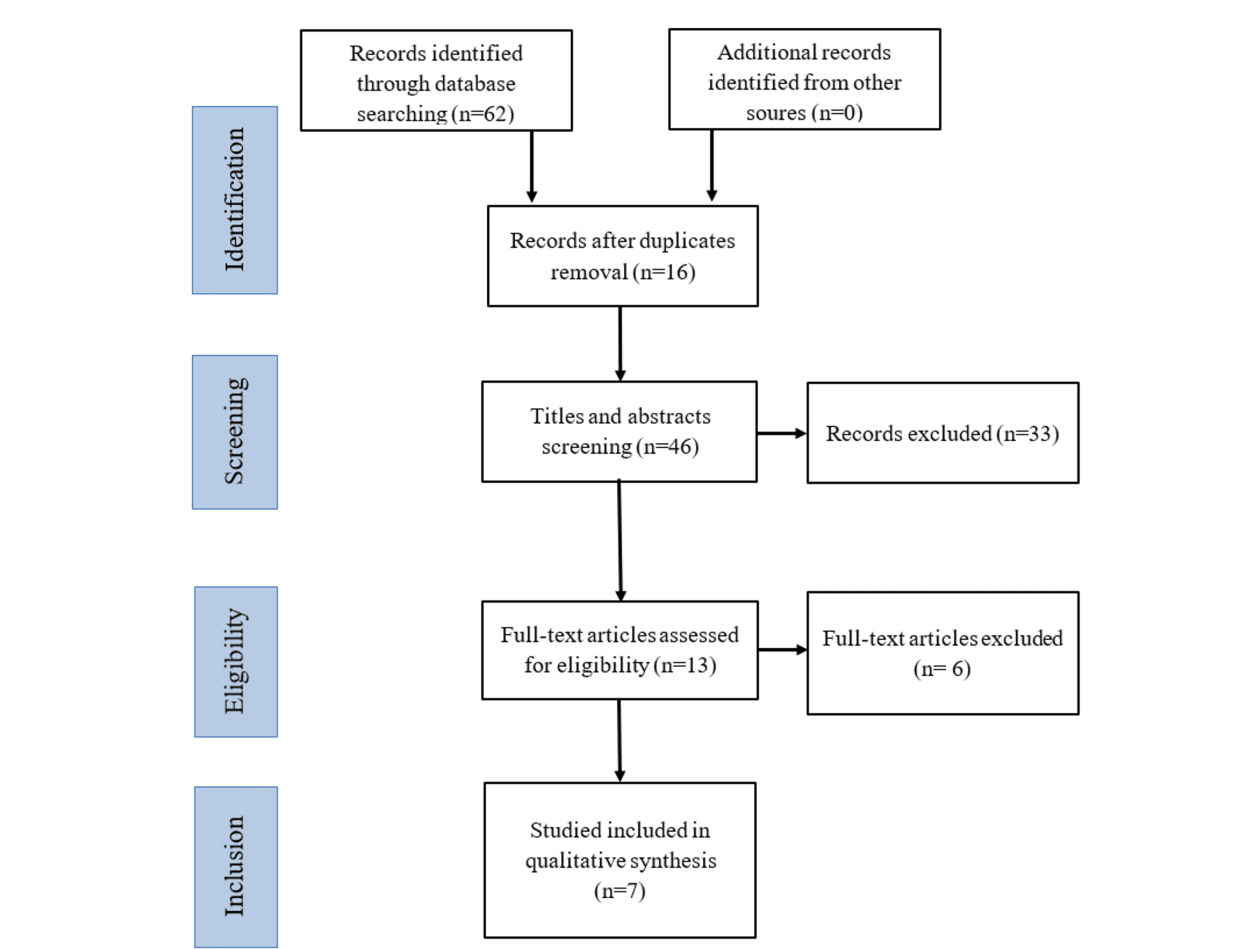
PRISMA flow diagram of studies screening and selection.

A total of 62 relevant studies were identified from all databases (PubMed, ScienceDirect, and Cochrane). After removing duplicates, 46 studies were screened for title/abstract evaluation. Following the inclusion criteria, 13 studies were selected for the full-text screening phase. Subsequently, six studies were excluded for the following reasons: absence of endodontic treatment on primary teeth (n=2), not describing the results of root canal treatment of primary teeth using chlorhexidine gluconate (n=4). Seven studies fulfilled all selection criteria and were included in the qualitative analysis. Figure 1 illustrates the screening and selection process.

Studies Characteristic

Children in the studies were aged 3-12 years, with 381 samples participating in seven studies (Table 1). Of these, six were clinical studies and one was an in vitro study using scanning electron microscopy (SEM). Of the six clinical studies, three were randomized controlled clinical trials. The studies were published between 2003 and 2020. Regarding study locations, four studies were conducted in India (57.14%), one in Egypt, one in Mexico, and one in Turkey.

**Table 1:** Characteristics of studies. RCT- Randomized clinical trial *NM: Not mentioned.

	Authors	Year of publication	Location	Research type	Age	Irrigation volumes	Unit	Follow-up
1	Barakat et al. [7]	2020	India	Controlled clinical trial	3-5	NM*	%	3 days
2	Walia et al. [8]	2019	India	In vivo	3-10	15 ml	%	Immediately
3	Jolly et al. [9]	2013	India	RCT	6-12	2 ml	CFU/ml	3 days
4	Ruiz-Esparza et al. [10]	2011	Mexico	RCT	3-9	0.5 ml	CFU/ml	Immediately
5	Musani et al. [11]	2009	India	Uncontrolled clinical trial	4-6	NM*	CFU/ml	Immediately
6	Onçağ et al. [12]	2003	Turkey	Uncontrolled clinical trial	4-10	2 ml	%	3 days
7	Hariharan et al. [13]	2010	India	RCT		10 ml	Smear layer removal by Rome scale	Immediately

The studies used irrigation volumes ranging from 0.5 to 20 ml and did not mention irrigation times. Four studies were evaluated at two time points, before and immediately after irrigation and shaping. The remaining studies were assessed before and after three days.

Antibacterial Properties of CHX

For the antibacterial properties of CHX, six studies were analyzed. Five studies showed that CHX is superior to NaCl by 0.9%, as demonstrated in five studies (Table 2). Comparisons with NaOCl reveal discrepancies among the studies. In Walia's study using NaOCl 1%, the antibacterial properties of NaOCl 1% and 2% CHX were found to be equivalent, with no statistical significance (p=0.409) [8]. Musani et al.'s research showed that the antibacterial efficacy of NaOCl 3% was inferior to 2% CHX [11]. In the case of NaOCl 5.25%, Oncag et al. suggested that the resistance against aerobic strains was similar, but 2% CHX was more effective against anaerobic strains (p<0.05) [12]. A controlled clinical trial by Barakat et al. (2020) on 60 primary incisors demonstrated that the antibacterial efficacy against E. faecalis of both solutions was comparable, with no statistically significant difference [7].

**Table 2:** Effectiveness of bacteria reduction in different irrigation groups. *NM: Not mentioned.

	Author	Year of publication	Comparison group	Sample size	Type of test	Results			Conclusion
						Initial value	Post irrigation value	Observations	
1	Barakat et al. [7]	2020	0.5% Metronidazole	20	Reduction quantity of E. faecalis units (%)	0	91.4%	There is no significant difference between the effects of CHX 2.0% and metronidazole 0.5% on E. faecalis, while there is a significant difference when compared to normal saline.	Both metronidazole 0.5% and 2% CHX demonstrate superior antibacterial effectiveness against E. faecalis compared to saline.
			2% CHX	20		0	96.1%		
			9% NaCl	20		0	49.1%		
2	Walia et al. [8]	2019	2% CHX	15	Reduction number of bacterial units CFU/ml (%)	0	96.62%	2% CHX, NaOCl 1%, and diode laser significantly reduce the percentage of bacterial units (statistical significance).	All three research groups successfully achieved a significant reduction in the bacterial load within the root canal compared to baseline.
			1% NaOCl	15		0	93.52%		
			Laser diode	15		0	84.20%		
			0.9% NaCl	15		0	56.83%		
3	Jolly et al. [9]	2013	NaCl 0.9%	15	Reduction number of anaerobic and aerobic bacteria (CFU/ml)	Aerobic: 7.13 ± 1.19 Anaerobic: 7.4 ± 1.24	Aerobic: 3.8 ± 1.08 Anaerobic: 6.4 ± 0.91	Antibacterial activity against aerobic strains: NaCl ~ CHX > DMSO > Ca(OH)₂ Antibacterial activity against anaerobic strains: CHX > DMSO > Ca(OH)₂ > NaCl	2% CHX is superior in its antibacterial efficacy against both aerobic and anaerobic bacteria, followed by DMSO, while 4% Ca(OH)₂ demonstrates the least effectiveness.
			2% CHX	15		Aerobic: 7.13 ± 1.19 Anaerobic: 7.2 ± 1.26	Aerobic: 3.8 ± 1.08 Anaerobic: 3.73 ± 0.88		
			Ca(OH)_2_ 4%	15		Aerobic: 7.13 ± 1.19 Anaerobic: 7.13 ± 1.19	Aerobic: 5.73 ± 1.49 Anaerobic: 5.27 ± 1.58		
			Dimethyl sulfoxide (DMSO) extract of propolis (4%)	15		Aerobic: 6.93 ± 1.58 Anaerobic: 7.27 ± 1.28	Aerobic: 3.87 ± 1.19 Anaerobic: 4.4 ± 1.12		
4	Ruiz-Esparza et al. [10]	2011	2% CHX	20	Reduction number of bacterial units CFU/ml	1.5 x 10^9^ ± 5.2 x 10^7^	1.5 x 10^6^ ± 4.6 x 10^6^	Analysis showed a statistically significant difference for the 2% CHX group (P < 0.0001, Mann-Whitney U test)	2% CHX exhibits greater antibacterial activity in the root canal compared to sterile 0.9% NaCl. CHX shows promise as an alternative irrigation solution in the treatment of necrotic primary teeth.
			NaCl 0.9%	20		1.68 x 10^9^ ± 4.7 x 10^7^	1.06 x 10^9^± 4.6 x 10^7^		
5	Musani et al. [11]	2009	2% CHX + Protaper	10	Reduction number of bacterial units CFU/ml	NM*	462.5 ± 232	The detectable bacterial count after shaping with hand-held ProTaper and 2% CHX is the lowest compared to the other study groups.	Cleaning the root canal is a challenge due to its complex morphology and the level of bacterial contamination. Therefore, effective cleaning with mechanical agents is essential.
			NaOCl 3% + Protaper	10		NM*	5175 ± 5369.27		
			2% CHX + K file	10		NM*	4350 ± 2821.64		
			NaOCl 3% + K file	10		NM*	8350 ± 13470.33		
6	Onçağ et al. [12]	2003	CHX 0.2% + Cetrimide 0.2%	23	Reduction number of bacterial units CFU/ml (%)	NM*	Aerobic: 73.3% Anaerobic: 88.24%	All comparison groups showed a statistically significant reduction in the levels of both aerobic and anaerobic bacteria compared to the control group.	Cetrexidin and 2% CHX exhibit stronger antibacterial activity and lower toxicity than 5.25% NaOCl, therefore suitable as irrigant for endodontic treatment in primary teeth.
			NaOCl 5.25%	25		NM*	Aerobic: 76.92% Anaerobic: 55.56%		
			2% CHX	22		NM*	Aerobic: 100% Anaerobic: 77.78%		
			NaCl 0.9%	21		NM*	Aerobic: 11.1% Anaerobic: 0%		

Tissue Solubility of CHX

Hariharan et al.'s in vitro study on removing the smear layer found that 6% citric acid was more effective in eliminating the smear layer compared to 10% EDTA and 5.25% NaOCl. Both 2% CHX and 0.9% NaCl were ineffective in removing the smear layer (Table 3) [13].

**Table 3:** Tissue solubility in different irrigation groups.

Comparison group	Sample size	Rome scale	Results				Conclusions
			Cervical thirds	Middle thirds	Apical thirds	Observations	During the preparation process, the root canal can be irrigated with saline. The final irrigation should use 6% citric acid, followed by 2% CHX.
NaCl 0.9%	6	Rome Scoring System: 0 points: No smear layer, open dentinal tubules, no erosion. 1 point: No smear layer, open dentinal tubules, eroded. 2 points: More than 50% of tubules visible. 3 points: Less than 50% of tubules visible. 4 points: Thick smear layer present, tubules occluded.	3.67	3.5	3.83	- Citric acid 6%: No smear layer present, open dentinal tubules - CHX: Thick smear layer present, tubules occluded.	
NaOCl 5.25%	6		3.00	3.00	3.67		
EDTA 10% + NaOCl 5.25%	6		1.00	1.17	1.33		
Citric acid 6%	6		0.00	0.00	0.67		
2% CHX	6		3.67	3.83	4.00		

Discussion

CHX is widely recognized for its two main formulations: solution and gel. A comprehensive review of the literature reveals that the 2% CHX solution has been the focus of most studies, with findings suggesting that this formulation is more suitable for clinical use compared to its gel counterpart or other concentrations [14]. The efficacy of 2% CHX as an irrigant is highlighted by its potent antibacterial properties, which are crucial for effective root canal disinfection. However, it is important to note that the volumes of irrigation used in various studies were inconsistent, which may affect the comparability of their results [15]. Two studies did not provide details regarding the irrigation volume, leaving a gap in understanding how this variable influences outcomes [7, 11]. Other studies reported varying irrigation volumes, from 0.5 ml to 10 ml. This lack of standardization complicates efforts to draw definitive conclusions about the optimal volume of CHX solution needed for effective irrigation in endodontic procedures. Despite these inconsistencies, the research conducted by Onçağ et al. and Jolly et al. is noteworthy [9, 12]. The study of Onçağ et al. demonstrated a significant bacterial reduction of up to 100% after irrigation with just 2 ml of 2% CHX [12]. This finding suggests clinicians may consider using a minimum of 2 ml of CHX solution during irrigation for effective bacterial reduction. Moreover, variables such as irrigation time, the type of irrigation needle, and supportive irrigation techniques were not specified in the analyzed studies. Future investigations on these factors could provide valuable insights to enhance clinical practices and improve patient outcomes in pediatric endodontics.

Antibacterial Activity

The antibacterial properties of CHX can be attributed to its positively charged cationic structure. This molecular feature allows CHX to interact effectively with the negatively charged phosphates in bacterial cell membranes. When used at a concentration of 2%, CHX demonstrates strong bactericidal effects by precipitating the cytoplasm of bacterial cells, disrupting their metabolic processes, and ultimately leading to cell death. This mechanism of action is particularly advantageous in endodontic procedures, where bacterial elimination is essential for successful treatment. Research by Oncag has shown that CHX is more effective against anaerobic bacterial strains than 5.25% sodium hypochlorite (NaOCl) [12]. In comparative studies, CHX achieved a bacterial reduction of 77.8%, while NaOCl reduced only 55.6%. This enhanced efficacy of CHX is especially relevant in pediatric dentistry, where anaerobic bacteria are commonly found in the necrotic pulp of primary teeth, as highlighted in Lemos' study (2020) [16]. These bacteria pose significant challenges for successful disinfection and treatment outcomes. While NaOCl is widely known for its ability to dissolve residual pulp tissue and its antibacterial properties, its use in primary teeth can lead to complications, such as sensitivity, irritation, or even severe damage to periodontal tissues, including the gums, mucosa, and periapical tissues. Based on these considerations, Thakur et al. (2020) suggested that chlorhexidine gluconate may be a safer and more effective irrigation option in necrotic pulp cases [5]. By minimizing the risk of tissue damage while maximizing antibacterial efficacy, CHX proves to be a preferable choice for pediatric endodontic treatments.

The clear advantages of CHX, particularly its effectiveness against anaerobic bacteria and its lower toxicity profile compared to sodium hypochlorite, make it a valuable tool for dental practitioners. Future research and clinical guidelines should further explore its role and optimize its use in endodontic procedures for primary teeth, ultimately improving patient care and treatment outcomes in pediatric dentistry.

Tissue Solubility

Hariharan et al.’s study demonstrated that CHX cannot clean inorganic tissue [13]. However, a recent study has offered a new perspective on using CHX as a sole irrigation solution. A randomized clinical trial by Zandi et al. (2019) compared the efficacy of 1% NaOCl and 2% CHX as the sole irrigants for 52 retreatment cases [17]. The clinical healing outcomes after one year were 65% and 64%, respectively (with no statistically significant difference), and after four years, the results were 81% and 82%, again with no significant difference. This raises the question of whether CHX can fully clean the root canal and whether combining it with other irrigation solutions or methods might be necessary to achieve optimal results.

Toxicity

Although many studies focusing on permanent teeth have demonstrated that chlorhexidine (CHX) is significantly less toxic than sodium hypochlorite (NaOCl), there is a lack of research concerning the potential effects of CHX on the periapical tissues of primary teeth [12, 18, 19]. The absence of studies specifically addressing the toxicity of CHX in the context of primary teeth is concerning, as the potential adverse effects on developing tissues may differ significantly from those observed in permanent teeth. Given the critical role of periapical tissues in maintaining dental health, it is essential to understand how CHX interacts with these tissues during endodontic treatments in primary teeth.

## Conclusions

Chlorhexidine gluconate in a 2% solution has demonstrated high antibacterial efficacy, particularly against aerobic bacteria. However, its effectiveness against anaerobic bacteria has not been as clearly studied. Although CHX cannot dissolve inorganic tissue, and no studies have yet determined its capacity to dissolve organic tissue, its strong antibacterial properties still make it a valuable option in endodontic treatment.

According to existing research, the minimum irrigation volume when using CHX is 2 ml per root canal, which ensures optimal effectiveness in bacterial elimination and improves treatment outcomes. However, to gain a deeper understanding of CHX’s capabilities, further studies are needed to clarify other aspects of this solution, particularly its ability to dissolve organic tissue and its effects on surrounding tissues in treating primary teeth.
